# Intelligence

**Published:** 1829-03

**Authors:** 


### INTELLIGENCE.

### MONTHLY REPORT OF PREVALENT DISEASES.

DURING the course of the last month, scarlet fever has been very prevalent. In most cases the diseases has been more than usually severe, and in a few instances children have fallen victims to it, the symptoms from the commencement having indicated danger. In these cases the disease ran a very rapid course, destroying the patient in four or five days from the attack. Its chief characteristics were, from the beginning, fever of a very low type, a small and indistinct pulse, disposition to coma, extensive ulcerations in the throat, with dark sloughs. As commonly happens where the throat is thus severely affected, the eruption upon the skin was slight; the cutaneous efflorescence being more apparent in the milder cases. In one instance, the patient, an adult female, laboured under the above symptoms, together with slight delirium. Dr. MACLEOD prescribed for her saline draughts with an excess of ammonia, and she evidently derived much benefit from the remedy. Her skin became of a more regular temperature; slight perspirations took place; the delirium ceased ; and in a few days she was convalescent.

---

J. HOULTON, Esq. F.L.S., at a recent meeting of the Medico-Botanical Society, exhibited a specimen of the extract of the *Cheonopodium olidum.* Mr. H. has in two cases found this remedy to possess the most decided emmenagogue powers. By former writers on materia medica the same virtue has been ascribed to this plant, although it has lately been much, or perhaps entirely, neglected. When we consider the very scanty list of emmenagogue remedies we have, the propriety of not losing sight of the suggestion of Mr. Houlton must be evident.

---

*Royal College of Physicians.*—The first evening meeting took place at the College of Physicians on Monday evening, February 9th. The company was not numerous, and consisted principally of visitors. Two papers were read ; the first, by the late Dr. BAILLIE, on the subject of Paraplegia. It contained a brief recital of many cases of that form of palsy, and its principal object was to show that paraplegia was more frequently dependent upon disease of the head than of the spine. The second communication, by Dr. GREGORY, stated the result of his experience at the Small-Pox Hospital during the year 1828. It is decidedly favorable to the cause of vaccination.

*Société Royale de Bordeaux.*—A prize of three hundred francs has been offered by this Society for the best Treatise upon the following subject: "To describe Puerperal Peritonitis, and to determine, by clinical facts, the particular cases in which the various modes of treatment that have been recommended are to be applied." Papers to be written either in Latin or French, and addressed (before the 15th June, 1829, free of expense,) to M. Dupuch Lapointe, secretary of the Society, Rue de la Grande Taupe, No. 21, Bordeaux.

---

*Surgical Lectures at St. Bartholomew's.*—In consequence of the severe indisposition of Mr. ABERNETHY, Mr. LAWRENCE has been appointed to give the surgical course at St. Bartholomew's Hospital.

---

*Royal Universal Infirmary for Children.*—Mr. DOUBLEDAY has been elected surgeon to this institution.

---

CÆSAR HAWKINS, Esq. has been elected surgeon of St. George's Hospital; a vacancy having occurred in consequence of the lamented death of Mr. ROSE.

---

Mr. LISTON, of Edinburgh, has lately published a letter, denying that he is the author of the "Lectures on Aneurism" which were some time ago published in the Lancet, and attributed to him!

---

*Physiology.*—Mr. BROUGHTON, surgeon to the 2d Life Guards, and one of the surgeons of the St. George's and St. James's Dispensary, has recommenced his Physiological Lectures to the dispensary pupils and others, who have gratuitous admission to them, on application at the house of the charity, in King street, Golden square, every Tuesday and Thursday evening, from eight to nine o'clock. These lectures have been well attended, and we have had very favorable accounts of them from those who are very capable of judging of their merit.

---

*False Affidavits made at Apothecaries' Hall by Candidates.*—It has been rumoured, but we hope without foundation, that it is common for false certificates to be presented to the Apothecaries' Company by *gentlemen* who present themselves for examination. For the honour of the profession, we trust such an occurrence is at least rare. If it is presumed that such certificates are carelessly investigated, it is a great mistake.

We have no wish to add to the punishment which has been already inflicted, by making the names of certain parties still more notorious than they already are; but it may be proper to mention, that in one case a true bill has been found for a misdemeanour, in consequence of the candidate having made a false affidavit of his age; and in another instance six months' imprisonment has been awarded, for having presented false indentures of apprenticeship.

### LITERARY NOTICES.

*Pharmacopœia.*—We understand that a committee has been appointed by the College of Physicians, to prepare a new edition of the Pharmacopœia.—The general utility of the work would be much increased, if the French and German names of drugs were given.

---

Dr. GRANVILLE's *Work, "On Abortion, and the Diseases of Menstruation."*—For many years Dr. G. has been collecting materials and arranging the results of his extensive experience upon these subjects, and we are happy to find that his work is now in a forward state of preparation. We have been favored with a view of some of the illustrative plates and drawings, which have been executed by PERRY in a very masterly and correct manner.

Upon the subject of abortion we have as yet only very imperfect essays. We have every reason to believe, from the numerous records of cases we have seen in the possession of Dr. G., that his work will be rich in practical facts, and that it will completely fill up the blank in the English catalogue of medical works which we have hitherto had to lament.

---

We are glad to find that a third edition of MASON GOOD'S "*Study of Medicine*," containing all the author's final corrections and improvements, together with additional modern information on physiology, practice, pathology, and the nature of diseases in general, is now preparing for publication by Mr. SAMUEL COOPER, author of the Dictionary of Practical Surgery, &c. From the extraordinary diligence and great judgment Mr. Cooper has shown in his previous publications, we have reason to congratulate the profession upon his having undertaken this task.

---

Mr. DUFFIN "*On the Influence of Physical Education in producing and confirming, in Females, Deformity of the Spine.*"—Although this volume is intended rather for popular than professional reference, it will be found to contain many hints that are not unworthy the attention of the medical practitioner. The author has not been so desirous of giving an air of novelty to his performance, as of collecting, and illustrating from his own experience, various facts which are scattered through many volumes, in proof of the serious injury inflicted upon young females by the common errors of modern education. The most likely modes of preventing the serious evils which too frequently result from an injudicious system of school discipline are pointed out, and many judicious observations are offered upon the various kinds of corporeal exercise which should be employed by young females for the preservation of their health, as well as for their recreation.

---

MAYO'S "*Outlines of Human Physiology.*"—A second edition of this valuable work has recently been published. Every part of it has been revised, and to many chapters much additional information is added. The important subject of the functions of the nerves, which has been so materially elucidated by the physiological experiments of Mr. Mayo, is treated at much greater length than in the former edition. It is frequently difficult, if not impossible, to convey to the mind of the student, by verbal description, a clear conception of the structure of parts: Mr. Mayo has therefore very judiciously added to the present edition many illustrative sketches, which the pupil in physiology will find of great assistance to him. We know no book in which the elements of physiology are more perspicuously and usefully described.

---

"*The Pupil's Introduction to Botany; by* JOHN STEGGALL, M.D."—The recent regulations of the College of Surgeons, and the Company of Apothecaries, demand from the student a certain extent of botanical knowledge. In this little volume he will find, in a very cheap form, every requisite elementary information upon the subject. The general reader, also, who is about to enter upon botanical studies, cannot select a more effectual assistant to clear his path, than this brief but instructive book. A list of plants in the London, Edinburgh, and Dublin Pharmacopœias, is given; and a very copious glossary of botanical terms is also appended to the volume. The plates will be found of much assistance to the pupil in botany.

### METEOROLOGICAL JOURNAL,

By Messrs. HARRIS and Co. Mathematical Instrument Makers, 50, High Holborn.

| January | Rain gauge. | Moon. | Thermom. | | | Barometer. | | De Luc's Hygrom. | | Winds. | | Atmospheric Variations. | | |
|---|---|---|---|---|---|---|---|---|---|---|---|---|---|---|
| | | | 9 A.M. | MAX. | MIN. | 9 A.M. | 10P.M. | 9A.M. | 10P.M. | 9 A.M. | 10P.M. | 9 *a.m.* | 2 *p.m.* | 10 *p.m.* |
| 20 | | ○ | 28 | 32 | 27 | 29.94 | 29.86 | 65 | 65 | NNE | NNE | Fog | Foggy | Fine |
| 21 | | | 29 | 29 | 22 | .81 | .76 | 65 | 63 | NE | ENE | F.& Sn. | Snow | Cloudy |
| 22 | | | 25 | 30 | 25 | .57 | .54 | 63 | 60 | E | E | Fine | Fine | Fine |
| 23 | | | 25 | 22 | 19 | .44 | .41 | 60 | 63 | E | ENE | Fine | Snow | Seow |
| 24 | | | 20 | 27 | 20 | .43 | .52 | 63 | 64 | N | NW | Fine | Fine | Cloudy |
| 25 | | | 20 | 23 | 21 | .50 | .38 | 65 | 65 | NNE | NNE | Foggy | Foggy | Fine |
| 26 | | | 32 | 42 | 35 | 28.97 | .02 | 70 | 70 | SE | SSW | Foggy | Fine | Fine |
| 27 | | | 38 | 41 | 35 | 29.01 | .04 | 70 | 69 | SSW | SSW | Cloudy | Rain | Fine |
| 28 | .03 | ☾ | 38 | 41 | 35 | .27 | .49 | 69 | 67 | W | W | Foggy | Fine | Cloudy |
| 29 | | | 36 | 40 | 33 | .43 | .37 | 68 | 70 | WNW | E | Fog | Cloudy | Fine |
| 30 | | | 35 | 40 | 34 | .42 | .67 | 69 | 69 | NE | NW | Fine | Fine | —— |
| 31 | | | 37 | 39 | 33 | 30.01 | 30.20 | 67 | 64 | N | NE | Fine | —— | —— |
| Feb. | | | | | | | | | | | | | | |
| 1 | | | 33 | 39 | 25 | .28 | .40 | 60 | 60 | NE | SE | —— | —— | —— |
| 2 | | | 27 | 33 | 24 | .43 | .44 | 58 | 58 | SE | S | Foggy | Fine | —— |
| 3 | | | 26 | 40 | 26 | .46 | .44 | 58 | 60 | S | SW | Fog | —— | —— |
| 4 | | ● | 35 | 45 | 35 | .28 | .14 | 60 | 69 | W | W | Foggy | Rain | Rain |
| 5 | .07 | | 45 | 41 | 38 | .03 | .19 | 69 | 69 | NW | WNW | Foggy | Cloudy | Cloudy |
| 6 | | | 40 | 43 | 40 | .22 | .10 | 70 | 70 | NW | NW | Fogg | Sleet | Cloudy |
| 7 | | | 43 | 46 | 38 | .03 | .02 | 70 | 68 | WNW | WNW | Cloudy | Cloudy | Snow'ry |
| 8 | .02 | | 39 | 40 | 35 | .13 | .22 | 67 | 67 | E | NW | Cloudy | Cloudy | Cloudy |
| 9 | | | 40 | 43 | 37 | .20 | .20 | 67 | 65 | W | N | Fogg | Fine | Cloudy |
| 10 | | ☽ | 38 | 42 | 38 | .30 | .21 | 65 | 66 | NSE | SE | Fine | Fine | Fine |
| 11 | | | 41 | 42 | 37 | .21 | .19 | 67 | 69 | WSW | WNW | Rain | Cloudy | Cloudy |
| 12 | | | 39 | 41 | 42 | .15 | .06 | 69 | 70 | WNW | WSW | Cloudy | Sleet | Sleet |
| 13 | .03 | | 45 | 48 | 39 | .03 | .03 | 71 | 65 | NW | NW | Sleet | Fine | Cloudy |
| 14 | | | 44 | 47 | 43 | .03 | 30.00 | 62 | 60 | W | WSW | Foggy | Fine | Fine |
| 15 | | | 46 | 50 | 45 | 30.00 | 29.95 | 64 | 64 | SW | SW | Fine | Cloudy | Cloudy |
| 16 | | | 45 | 51 | 41 | 29.87 | .76 | 65 | 65 | SW | SW | Foggy | Fine | Cloudy |
| 17 | | | 43 | 48 | 36 | .65 | .75 | 65 | 64 | W | ESE | Fine | Fine | Fine |
| 18 | | ○ | 38 | 39 | 36 | .74 | .70 | 64 | 63 | ESE | SEva. | —— | —— | Cloudy |
| 19 | | | 39 | 48 | 39 | .55 | .45 | 63 | 65 | SE | SSW | —— | —— | Fine |

The quantity of Rain fallen in the month of January cannot be given, the Rain-gauge having been frozen.

